# Insights into the pathological mechanisms of p85α mutations using a yeast-based phosphatidylinositol 3-kinase model

**DOI:** 10.1042/BSR20160258

**Published:** 2017-03-15

**Authors:** María D. Oliver, Teresa Fernández-Acero, Sandra Luna, Isabel Rodríguez-Escudero, María Molina, Rafael Pulido, Víctor J. Cid

**Affiliations:** 1 Dpto. de Microbiología II, Facultad de Farmacia, Universidad Complutense de Madrid and Instituto Ramón y Cajal de Investigaciones Sanitarias (IRyCIS), 28040 Madrid, Spain; 2 Centro de Investigación Príncipe Felipe, 46012 Valencia, Spain; 3 Biocruces Health Research Institute, 48903 Barakaldo, Spain; 4 IKERBASQUE, Basque Foundation for Science, 48013 Bilbao, Spain

**Keywords:** humanized yeast, oncogenic mutations, Phosphatidylinositol 3-kinase, p85, p110, SHORT disease

## Abstract

In higher eukaryotes, cell proliferation is regulated by class I phosphatidylinositol 3-kinase (PI3K), which transduces stimuli received from neighboring receptors by local generation of PtdIns(3,4,5)*P*_3_ in cellular membranes. PI3K is a heterodimeric protein consisting of a regulatory and a catalytic subunit (p85 and p110 respectively). Heterologous expression of p110α in *Saccharomyces cerevisiae* leads to toxicity by conversion of essential PtdIns(4,5)*P*_2_ into futile PtdIns(3,4,5)*P*_3_, providing a humanized yeast model for functional studies on this pathway. Here, we report expression and functional characterization in yeast of all regulatory and catalytic human PI3K isoforms, and exploitation of the most suitable setting to functionally assay panels of tumor- and germ line-associated PI3K mutations, with indications to the limits of the system. The activity of p110α in yeast was not compromised by truncation of its N-terminal adaptor-binding domain (ABD) or inactivation of the Ras-binding domain (RBD). In contrast, a cluster of positively charged residues at the C2 domain was essential. Expression of a membrane-driven p65α oncogenic-truncated version of p85α, but not the full-length protein, led to enhanced activity of α, β, and δ p110 isoforms. Mutations impairing the inhibitory regulation exerted by the p85α iSH2 domain on the C2 domain of p110α yielded the latter non-responsive to negative regulation, thus reproducing this oncogenic mechanism in yeast. However, p85α germ line mutations associated with short stature, hyperextensibility of joints and/or inguinal hernia, ocular depression, Rieger anomaly, and teething delay (SHORT) syndrome did not increase PI3K activity in this model, supporting the idea that SHORT syndrome-associated p85α mutations operate through mechanisms different from the canonical disruption of inhibitory p85–p110 interactions typical of cancer.

## Introduction

Synthesis of PtdIns(3,4,5)*P*_3_ by phosphatidylinositol 3-kinase (PI3K) physiologically occurs in cellular membranes in response to the stimulation of growth factor receptors and other stimuli. PtdIns(3,4,5)*P*_3_-responsive downstream effectors of PI3K include, sequentially, phosphoinositide-dependent kinase (PDK), Akt, and mTor protein kinases, involved in multiple functions related to transcriptional regulation and protein synthesis [1–3]. Activation of these pathways ultimately promotes cell proliferation and growth, cell motility, and inhibition of programmed cell death [4]. Deregulation and hyperactivation of PI3K signaling is a landmark of multiple pathologies, especially cancer. The most common mutations leading to enhanced PtdIns(3,4,5)*P*_3_-dependent signaling map in the phosphatase and tensin homolog deleted on chromosome ten (PTEN) phosphoinositide 3-phosphatase, a prominent tumor suppressor [5–8]. Somatic gain-of-function mutations in the coding region of both PI3K subunit genes, *PIK3R1* (coding for p85α, the regulatory subunit of isoform α class I PI3K heterodimer) and *PIK3CA* (encoding the p110α catalytic subunit), are commonly found in many tumor types [9–12]. Such point mutations often appear in hotspots that correspond to structural regions that account for fine allosteric regulation of p110 by p85, such as those involved in the inhibitory interaction between the SH2 domains of p85 and p110 (reviewed in [13]). Archetypal examples of such oncogenic mutations are (i) the truncated p65α version of p85α, isolated from a murine lymphoma [14], which lacks the C-terminal SH2 (cSH2) domain, thus presumably affecting the inhibitory function mediated by the preceding N-terminal and intermediate SH2 domains (nSH2 and iSH2); or (ii) the p110α E545K point mutant in the helical domain [15], which relieves such inhibitory interaction [16]. Although crystallographic studies have revealed the interaction surfaces of the p85–p110 complex, many mechanistic questions about the regulation imposed on p110 by p85 remain unsolved.

The application of the latest analytical technologies to cancer genomics, such as single cell whole exome sequencing performed on tumors by The Cancer Genome Atlas (TCGA) project and others, is revealing a plethora of mutations in these oncogenic pathways [17–19]. Addressing whether such mutations have an influence in the function of these oncoproteins and by which molecular mechanisms they operate is currently an important scientific challenge. *Saccharomyces cerevisiae* is an easily manipulatable and genetically tractable model organism for molecular studies on heterologously expressed proteins. We have previously developed a ‘humanized yeast’ model by heterologous expression of the mammalian PI3K catalytic subunit (p110α) artificially driven to yeast membranes by a C-terminal prenylation box [20], and thoroughly exploited it for functional analyses of mutations in the tumor suppressor PTEN [21–25]. The model relies on the fact that budding yeast lacks PtdIns(3,4,5)*P*_3_-dependent pathways, but expression of the mammalian class I PI3K severely depletes its substrate PtdIns(4,5)*P*_2_ from the plasma membrane. The latter phosphoinositide is essential for polarized secretion and endocytosis in yeast, so its elimination by *in vivo* PI3K activity is reflected as growth inhibition [20,24].

Here, we explore the applicability of this yeast setting to perform functional studies on PI3K by co-expression of its regulatory and catalytic subunits. We used a set of mutants to evaluate the aspects and define the limits of PI3K regulation that can be assayed in this heterologous model. We found that the key features related to oncogenesis, namely PI3K gain-of-function by p85-dependent recruitment to the plasma membrane, and disruption of inhibitory p85–p110 interactions can be traced in yeast. Moreover, p85α mutations associated with the non-oncological short stature, hyperextensibility of joints and/or inguinal hernia, ocular depression, Rieger anomaly, and teething delay (SHORT) syndrome [26–28] behaved differently in our model as compared with those purportedly oncogenic, suggesting distinct pathological mechanisms for tumor- and germ line-associated p85α mutations.

## Results

### Involvement of distinct p110 domains in intrinsic PI3K activity in the heterologous yeast model

**Figure 1:**
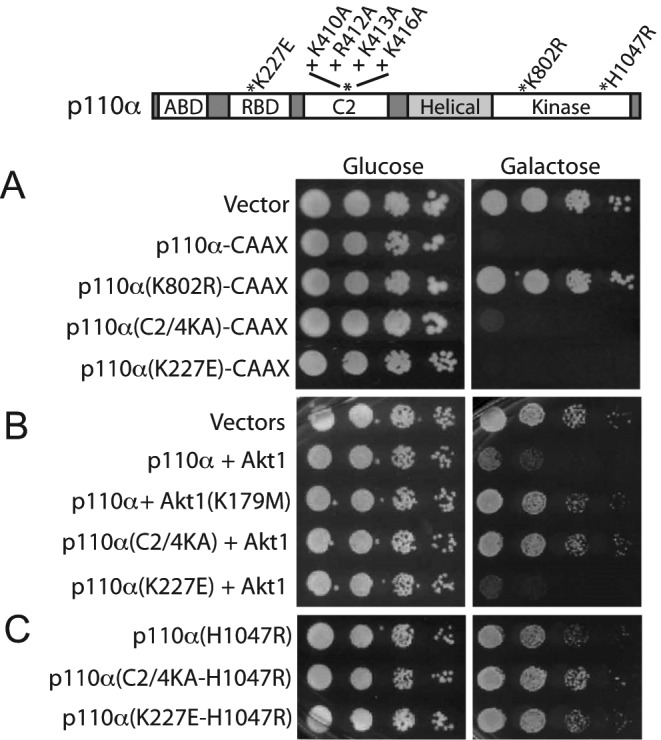
Contribution of C2 and RBD domains to p110α activity in yeast (**A**) 10-fold serial dilutions of wild-type YPH499 yeast transformed with empty YCpLG (vector) as growth control for plasmids, YCpLG–p110α–CAAX, YCpLG–p110α (K802R)–CAAX, YCpLG–p110α (C2/4KA)–CAAX, and YCpLG–p110α (K227E)–CAAX, as indicated. (**B**) Growth assay of cells co-transformed with YCpLG and pYES2 (vectors) or pYES2–GFP–Akt1 with YCpLG–p110α, pYES2–GFP–Akt1 (K179M; kinase-dead) with YCpLG–p110α, and pYES2–GFP–Akt1 with YCpLG–p110α (C2/4KA) or with YCpLG–p110α (K227E), as indicated. (**C**) Drop agar growth assay on yeast transformed with YCpLG–p110α (H1047R), YCpLG–p110α (C2/4KA)–H1047R, and YCpLG–p110α (K227E)–H1047R, as indicated. Yeast cells suspensions were spotted on synthetic complete (SC) medium, lacking the appropriate auxotrophic selection plasmid markers with either glucose (repression conditions) or galactose (for *GAL1*-driven expression) as carbon sources, respectively, as indicated. A representative experiment is shown from triplicate transformant clones assayed. Plates were incubated for 48 (glucose) or 72 h (galactose) at 28°C. A p110α primary structure sketch on top is provided as a reference to locate the mutations tested in (A)–(C) panels.

In previous studies, we developed a humanized yeast system by heterologously expressing p110, Akt, and PTEN isoforms [20,24,29]. Within that frame, we described that, when overproduced from the strong *GAL1* promoter (induced in galactose as a carbon source), p110α and β isoforms led to severe and mild, respectively, growth inhibition in yeast. This required their expression as membrane-directed proteins by attachment of an H-Ras C-terminal prenylation box (p110α–CAAX and p110β–CAAX), and was dependent on p110 catalytic activity (Figure 1) [20,21]. Although p110α did not inhibit yeast growth by itself in the absence of this membrane attachment signal, co-expression of native p110α and Akt isoforms led to growth inhibition by a different mechanism that relied on the activity of the Akt kinase [29]. However, the oncogenic mutation H1047R of p110α did show an evident degree of inhibition, thus reproducing its intrinsic hypermorphic phenotype [21]. In order to gain insight on the significance of both the C2 and Ras-binding domain (RBD) domains on p110α kinase activity by using the yeast model, we generated point mutations that potentially inactivate such domains in all three p110α–CAAX, p110α–H1047R, and p110α + Akt1 yeast experimental settings.

C2 domains bear structural motifs related to interaction with membrane phospholipids. It has been suggested that a cluster of positive residues in the p110α C2 domain (K410, R412, K413, and K416) was involved in its interaction with the plasma membrane through electrostatic interactions [30]. Therefore, we simultaneously mutated these four residues to alanine to locally reduce such positive charges. The resulting p110α–(K410A, R412A, K413A, K416A) will be referred hereafter as p110α–C2/4KA. As shown in Figure 1A, p110α–C2/4KA–CAAX kept its ability to strongly inhibit yeast growth, indicating that kinase activity was not impaired by the quadruple C2 mutation. However, p110α–C2/4KA fully lost its ability to activate Akt in yeast in the absence of a membrane-targeting prenylation CAAX signal (Figure 1B), proving that membrane recognition by the C2 domain is essential for p110α performance. Moreover, the C2/4KA mutations suppressed hyperactivation by the H1047R mutation (Figure 1C), denoting that the function of the C2 domain contributes to the enhanced activity of this allele.

In mammalian cells, interaction of p110 with the Ras oncoprotein via its RBD domain reinforces its recruitment to the plasma membrane thus contributing to PI3K activation [31]. Yeast possesses two Ras homologs, Ras1 and Ras2 [32], which could be playing this role in activation of heterologous p110. To test whether RBD function was determinant for p110α activity in yeast, we introduced the K227E mutation, which abolishes the interaction with Ras in mammalian cells [33], in the same expression settings as above. In all cases, the K227E mutant behaved as the equivalent p110α version with a wt RBD (Figure 1A–C). These results imply that RBD function is not necessary for inherent p110α kinase activity *in vivo*.

**Figure 2:**
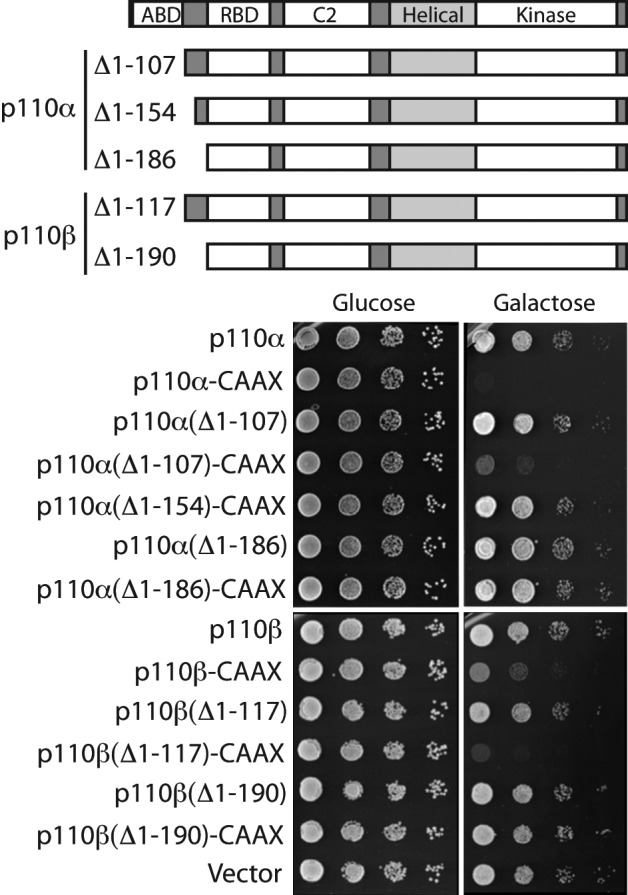
Functional analysis of p110α and p110β N-terminal truncations in yeast Ten-fold serial dilutions of YPH499 yeast transformants bearing the indicated plasmids, as indicated: YCpLG–p110α, YCpLG–p110α–CAAX, YCpLG–p110α(∆1-107), YCpLG–p110α(∆1-107)–CAAX, YCpLG–p110α(∆1-154)–CAAX, YCpLG–p110α(∆1-186), YCpLG–p110α(∆1-186)–CAAX, YCpLG–p110β, YCpLG–p110β–CAAX, YCpLG–p110β(∆1-117), YCpLG–p110β(∆1-117–CAAX), YCpLG–p110β(∆1-190), YCpLG–p110β(∆1-190–CAAX) and an empty vector (YCpLG). Yeast cell suspensions were processed as in Figure 1. Sketches of p110α and p110β primary structures indicating the missing residues in the assayed different truncations are shown on top.

The interaction between the regulatory subunit p85 iSH2 domain and the N-terminal adaptor-binding domain (ABD) of p110 facilitates interaction with the plasma membrane [16], triggering the signaling cascade. In yeast, membrane-targeted p110 is active in the absence of p85, so we could address, by generating N-terminal truncations, whether these domains were structurally necessary for intrinsic p110 PI3K activity *in vivo*. Hence, we generated a series of N-terminal-truncated versions of both p110α and β isoforms, as shown in Figure 2. For p110α we generated truncations lacking the ABD domain (p110α ∆1-107), the ABD domain and a fragment of the ABD/RBD linker (p110α ∆1-154), and the ABD domain plus the whole ABD/RBD linker (p110α ∆1-186). For p110β we generated truncations lacking the ABD domain (p110β ∆1-117) and the ABD domain plus the whole linker ABD/RBD (p110β ∆1-190). In both cases, truncations were produced both in the absence and in the presence of a C-terminal prenylation signal. Plasma membrane-directed constructions lacking the ABD domain (p110α ∆1-107–CAAX and p110β ∆1-117–CAAX) still caused yeast growth inhibition (Figure 2), whereas those lacking ABD domain and the whole linker (p110α (∆1-186) and p110β (∆1-190)) had lost their activity. Moreover, partial loss of the linker was sufficient to cause activity loss in p110α (p110α (∆1-154)–CAAX). A p110α ∆1-108 deletion displayed decreased expression in mammalian cells [34], suggesting that larger N-terminal truncations could have compromised stability. In line with this, we could not detect expression of our N-terminal p110 truncations in yeast. Thus, the whole ABD is dispensable, but additional N-terminal deletions in p110 yield it inactive in yeast, likely by protein destabilization.

### Expression and function of PI3K regulatory subunits in yeast

**Figure 3:**
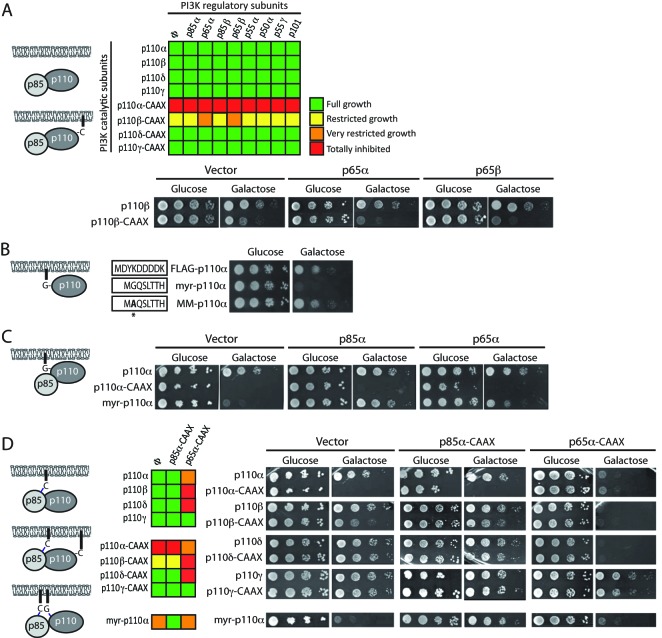
Effects of co-expression of p85 and p110 subunits and myristoylation of p110α (**A**) Expression of all class I PI3K regulatory subunits in yeast in combination with native or C-terminally prenylatable p110 subunits. Color-code graphical representation of the effect on yeast growth in galactose, assayed as in Figures 1 and 2, of the co-transformants bearing either empty pYES3/CT vector (ϕ) or plasmids bearing the regulatory subunits pYES3–p85α, pYES3–p65α, pYES3–p85β, pYES3–p65β, pYES3–p55α, pYES3–p50α, pYES3–p55γ, and pYES3–p101, in combination with plasmids YCpLG–p110α, YCpLG–p110β, YCpLG–p110δ, YCpLG–p110γ, YCpLG–p110α–CAAX, YCpLG–p110β–CAAX, YCpLG–p110δ–CAAX, and YCpLG–p110γ–CAAX as indicated. Green color indicates wild-type-like growth and yellow, orange, and red colors indicate different degrees of growth inhibition, as indicated in the legend. Below the grid, a representative experiment is shown, corresponding to the YCpLG–p110β or YCpLG–p110β–CAAX transformants combined with pYES3 (vector), pYES3–p65α, or pYES3–p65β. (**B**) Activation of p110α by myristoylation. Growth assay of serial dilutions of YCpLG–FLAG–p110α, YCpLG–Myr–p110α, and YCpLG–MM–p110α transformants, as indicated. On the left side, the amino acidic sequences of the corresponding extra N-terminal extensions are shown. (**C**) Inhibition of Myr–p110α by p85α. Growth assay on co-transformants bearing YCpLG–p110α, YCpLG–p110α–CAAX, or YCpLG–Myr–p110α in combination with empty pYES3/CT (vector), pYES3–p85α, or pYES3–p65α. (**D**) Modulation of p110 versions by C-terminal prenylation of wild-type and truncated p85α. Growth assay on transformants bearing the pYES3/CT empty plasmid (ϕ or vector) or pYES3–p85α–CAAX, pYES3–p65α–CAAX co-existing with plasmids YCpLG–p110α, YCpLG–p110α–CAAX, YCpLG–p110β, YCpLG–p110β–CAAX, YCpLG–p110δ, YCpLG–p110δ–CAAX, YCpLG–p110γ, YCpLG–p110γ–CAAX, or YCpLG–Myr–p110α, as indicated. Both a color-code grid (left; same code as in Figure 3A) and an equivalent representative experiment (right) are shown. All experiments were performed in the YPH499 yeast strain, following the same procedures as in Figures 1 and 2. On the left side of each panel, a graphical representation of the corresponding combinations assayed is shown. C-terminal prenylation is depicted by a Cys bridge (C); N-terminal myristoylation is marked as a Gly bridge (G).

To reconstruct in yeast a model that resembles mammalian PI3K activity and regulation, we extended our system by expressing PI3K regulatory subunits. For this purpose, we expressed HA-tagged versions of the p85α and β isoforms, in addition to a p65α oncogenic C-terminal truncation comprising amino acids 1–571 [14], its corresponding truncation in p85β, hereafter referred as p65β (comprising residues 1–568) as well as p55α, p50α, p55γ, and p101. Although the proteins were properly overproduced under galactose induction conditions, as determined by immunoblot with anti-HA antibodies, they did not interfere with yeast growth or led to obvious phenotypes, neither when expressed alone (results not shown) or in combination with p110α, β, δ, and γ isoforms (Figure 3A). None of the regulatory subunits did counteract the strong inhibitory activity of p110α–CAAX. However, when co-expressed with p110β–CAAX, both p65α and p65β C-terminal truncations enhanced its toxicity (Figure 3A), suggesting that they exerted an activating effect on p110β, in agreement with the reported oncogenic activity of p65α [14].

Unlike higher cells, *S. cerevisiae* lacks tyrosine phosphorylation as a prominent signaling event. Thus, it was expected that activation of p110 by p85-dependent recruitment was not naturally reproduced in yeast, imposing some limits to our analysis. Still, these results underscore the requirement of the recruitment of p110α and p110β to plasma membrane for its function. Since C-terminal prenylation is probably too strong as a membrane-directing signal for p110α to actually detect fine-tuning interactions with p85, we tested N-terminal myristoylation as an alternative p110α membrane-targeting strategy. As shown in Figure 3B and 3C, Myr–p110α caused a moderate but significant inhibition of yeast growth as compared with p110α–CAAX. This was not due to sheer modification of p110α N-terminal end [35], as the expression of a mutant in which the glycine subjected to myristoylation was mutated to Ala (MM–p110α), was innocuous, as was a FLAG-tagged p110α version (FLAG–p110) (Figure 3B). The observation that Myr–p110α expression led to an intermediate growth inhibition phenotype, as compared with those of p110α–CAAX and p110α, prompted us to evaluate the effect of p85α/p65α in these conditions. Interestingly, p85α down-regulated Myr–p110α activity in yeast (Figure 3C), suggesting that the inhibitory effect performed by the regulatory subunits in the absence of tyrosine-phosphorylated receptors could be reproduced in the yeast system. In contrast, we were not able to reproduce on Myr–p110α the positive regulation carried out by the oncogenic version p65α on p110β–CAAX.

Finally, we introduced a CAAX prenylation signal on p85α and p65α, thus mimicking their membrane attachment by activated receptor tyrosine kinases (RTKs) in higher cells. We evaluated their effects on both plasma membrane-anchored p110 isoforms (p110α–CAAX, Myr–p110α, p110β–CAAX, p110δ–CAAX, and p110γ–CAAX) and their corresponding soluble forms (p110α, p110β, p110δ, and p110γ). Remarkably, p65α–CAAX enhanced the activity of all the class IA catalytic subunits tested (p110α, p110β, p110β–CAAX, p110δ, and p110δ–CAAX) but not that of class IB (p110γ) (Figure 3D). In contrast, like non-prenylated p85, p85α–CAAX down-regulated Myr–p110α activity in yeast, and slightly reduced p110β–CAAX toxicity (Figure 3D). Together, our findings illustrate that the yeast system can be used to investigate diverse aspects of PI3K function, related to membrane targeting, enzyme allosteric regulation, and perhaps stabilization, which affect differentially to the distinct PI3K class IA catalytic subunits.

### Functional studies on p85α-dependent regulation of p110 in yeast

**Figure 4:**
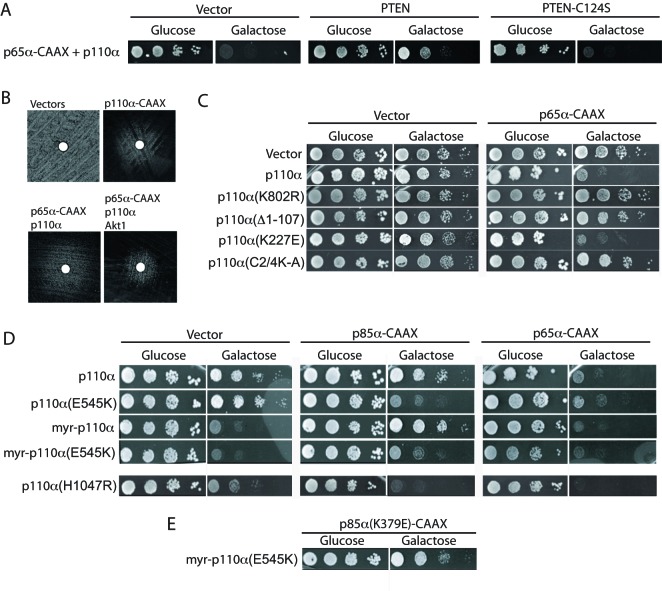
Characterization of p85–p110 functional interactions in yeast (**A**) Activation of p110 by p65 is counteracted by catalytically active PTEN. Growth of YPH499 triple transformants bearing pYES3–p65α–CAAX, YCpLG–p110α, and either pYES2 (vector), pYES2–PTEN, expressing the active phosphatase or pYES2–PTEN (C124S), expressing a phosphatase-dead PTEN mutant. (**B**) Halo growth assay on SC-galactose agar around a 6 mm-disk impregnated with the PI3K inhibitor 15e. A representative assay is shown on agar spread with Y03951 (*snq2*Δ) triple transformants bearing pYES2, pYES3/CT, and YCpLG (vectors, top left); pYES2, pYES3/CT, and YCpLG–p110α–CAAX (p110α–CAAX, top right); pYES2, pYES3–p65α–CAAX, and YCpLG–p110α (p65α–CAAX/p110α, bottom left); and pYES3–p65α–CAAX, YCpLG–p110α, and pYES2–GFP–Akt1 (p65α–CAAX/p110α/Akt1, bottom right). (**C**) The ABD and C2 domains are required for p110 activation by p65. Agar growth assays of YPH499 yeast co-transformed with either the pYES3 plasmid (vector) or pYES3–p65α–CAAX and the YCpLG empty plasmid (vector), YCpLG–p110α, YCpLG–p110α (K802R), YCpLG–p110α (∆1-107), YCpLG–p110α (K227E), or YCpLG–p110α (C2/4KA), as indicated. (**D**) Mutants affecting the allosteric regulation of p110 by p85 function in yeast. Growth assays of YPH499 co-transformed with the pYES3 plasmid (vector), pYES3–p85α–CAAX or pYES3–p65α–CAAX, in combination with YCpLG–p110α, YCpLG–p110α (E545K), YCpLG–Myr–p110α, YCpLG–Myr–p110α (E545K), and YCpLG–p110α (H1047R), as indicated. (**E**) Recovery of the inhibitory effect exerted by p85 on p110 by charge inversion in the nSH2/helical interface. Growth assay on a co-transformant bearing plasmids pYES3–p85α (K379E)–CAAX and YCpLG–p110α (E545K).

The above data show that the p65α–CAAX + p110α combination could be an appropriate setting to reproduce the positive effect of membrane recruitment of p110 by p85 in the absence of the inhibitory interaction imposed by full-length p85. Toxicity in yeast of the p65α–CAAX/p110α pair was dependent on its catalytic PI3K activity since (i) growth inhibition was counteracted by co-expression of PTEN (Figure 4A); (ii) it could be reverted by a p110 chemical inhibitor (Figure 4B); and (iii) a kinase-dead mutant p110α (K802R) was innocuous (Figure 4C). Moreover, the p65–CAAX/p110 combination was competent in activating co-expressed Akt1 leading to growth arrest, which could also be counteracted by a PI3K inhibiting compound (Figure 4B).

We then combined p65α–CAAX with mutants in different p110α domains. Co-expression with the p110α ∆1-107–truncated mutant showed that p65α–CAAX requires the presence of the p110α ABD domain to exert activating interaction (Figure 4C). Also, p110α–C2/4KA failed to become activated by p65α–CAAX, indicating that the ability of p110α itself to interact with the plasma membrane was determinant for an effective p65α-induced recruitment and activation (Figure 4C). In contrast, p65α–CAAX was fully competent activating the RBD-targeting p110α K227E mutation (Figure 4C).

Next, we used the p85α–CAAX to verify whether the negative regulation exerted by p85 on p110 in yeast involved inhibitory interaction between these two proteins. Indeed, the introduction of the oncogenic mutation E545K at the helical domain of p110α, which inverts the charge involved in electrostatic interactions with the nSH2 domain of p85α [36], released p110α from p85α–CAAX negative regulation: first, p85α–CAAX had an activating effect, compared with that of p65α–CAAX, on the p110α E545K mutant, but not on wild-type p110α (Figure 4D); and second, p85α–CAAX lost the ability to negatively regulate Myr–p110α when the E545K mutation was introduced in the latter (Figure 4D). Moreover, as described in higher cells [36], the introduction of a K379E mutation on the p85α nSH2 domain providing a complementary charge to that of p110α E545K was able to restore the inhibitory interaction. Thus, the combination of p85α K379E–CAAX with Myr–p110α E545K rescued yeast growth (Figure 4E). In conclusion, although our experiments do not directly measure protein–protein interactions, our results with the distinct combinations of mutations suggest that the yeast system recapitulates the p85–p110 allosteric regulatory interactions.

Finally, we tested whether the p110α H1047R mutant was sensitive to regulation by membrane-driven p85α. Interestingly, both p65α–CAAX and p85α–CAAX enhanced the activity of p110α H1047R (Figure 4D), suggesting that the conformation of this allele is sensitive in yeast to p85-driven membrane recruitment but insensitive to its inhibitory inputs.

### Enquiring cancer-related p85α mutations for function in the yeast system

**Figure 5:**
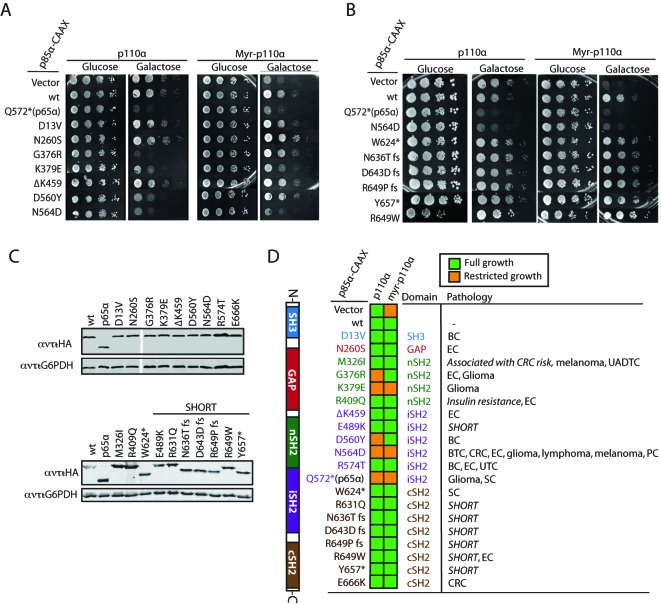
Functional analyses of p85α pathologic mutants in yeast Representative agar drop growth assays of selected YPH499 yeast transformant clones (**A** and **B**) and a graphical color-code display (**D**) of growth inhibition assays on all mutations tested by co-expression of the indicated combinations of plasmids: pYES3/CT (vector) or pYES3–p85α–CAAX (wt or bearing the indicated mutations or truncations) with YCpLG–p110α and YCpLG–Myr–p110α. (**A**) Selection of growth assays for clones expressing p85α mutations related to cancer. (**B**) Selection of growth assays for clones expressing p85α mutations related to SHORT disease, as well as a W624* truncation. N564D and p65α are shown as gain-of-function controls, as a reference. (**C**) Immunoblots showing the expression in yeast lysates of p85α mutants related to cancer or SHORT syndrome disease, as indicated. Membranes were hybridized with monoclonal anti-HA antibodies to detect N-terminally-tagged p85α variants and anti-G6PDH antibodies as a loading control. (**D**) A sketch of p85α primary structures and domains is shown (left). Blue, red, dark green, purple, and brown colors are used to depict localization of mutations to the different p85α domains. The pathology column relates mutations to the tumor type in which they were found (COSMIC database, http://cancer.sanger.ac.uk/cancergenome/projects/cosmic/) [52] or their linkage to SHORT syndrome [26–28]; BC, breast carcinoma; BTC, biliary tract carcinoma; CRC, colorectal carcinoma; EC, endometrium carcinoma; PC, prostate carcinoma; SC, stomach carcinoma; UADTC, upper aerodigestive tract carcinoma; UTC, urinary tract carcinoma; fs frame shift. Italics indicate germ line mutation.

The results above are suggestive that p85–p110 functional interactions can be traced in the yeast heterologous system. Thus, we chose a set of *PIK3R1* (encoding p85α) mutations to be expressed as p85α–CAAX, including N564D and a series of selected p85α point mutations isolated in tumors, some previously assayed *in vitro* or *in vivo* in mammalian cells, but most of them with a yet unclear mechanism [9,12,36–38]. We tested these mutants against both p110α and Myr–p110α. According to their behavior on the yeast system (Figure 5A and D), we were able to define three groups of mutations: (i) mutations that behave as the wild-type p85α–CAAX control, i.e. they exert a detectable inhibitory effect on Myr–p110α but do not have any effect on p110α (D13V, N260S, M326I, ΔK459, R574T, and E666K); (ii) a second group, conformed by K379E at the nSH2 domain and N564D at the iSH2 domain, behaved like p65α–CAAX, i.e. activating p110α and failing to inhibit Myr–p110α; and (iii) finally, mutations G376R (at nSH2) and D560Y (at iSH2) activated p110α but were able to inhibit Myr–p110α. The different performance of these sets of mutants was not biased by expression or stability in yeast since they were equivalently produced, as verified by Western blotting (Figure 5C).

### Germ line SHORT syndrome-related p85α mutations are competent for the inhibition of p110α

Next, we tested a panel of germ line *PIK3R1* mutations linked to the genetically inherited SHORT syndrome [39]. The panel covered most of the SHORT syndrome-associated *PIK3R1* coding region mutations currently identified, as well as *PIK3R1* mutations associated with colorectal cancer risk and with insulin resistance (HGMD^R^ Human Gene Mutation Database) (Figure 5B and D). All these mutations behaved indistinguishably from wild-type p85α for their ability to modulate p110α in yeast (Figure 5B and D), suggesting that functional p85α–p110α interactions were intact. Noticeably, most of the SHORT syndrome-linked mutations targeted a cSH2 residue or introduced a stop codon at the cSH2 domain, suggesting a dispensable role for the p85α cSH2 domain in the direct control of p110α activity. In accordance, a W624* truncation lacking the entire p85α cSH2 domain was also indistinguishable from wild-type p85α (Figure 5B and D). The different SHORT-related mutations were properly expressed in yeast, as verified by immunoblot (Figure 5C). Overall, we conclude that the functional pattern of SHORT syndrome-associated p85α mutations differs from the p110α-activating pattern manifested by either p65α or the most prevalent tumor-associated p85α human mutations.

## Discussion

In physiological conditions in higher eukaryotic cells, receptors activated by tyrosine phosphorylation recruit the p85–p110 PI3K heterodimer to the plasma membrane. The interaction between pYXXM domains in receptor/adaptor proteins and p85 SH2 domains releases inhibitory interactions between such domains and the C2/helical domains in the p110 catalytic subunit. This allosteric regulation allows a conformational switch in the PI3K heterodimer, leading to full activation of the kinase in the proximity of its phosphoinositide substrate [16,36,40,41]. Hypermorphic mutations in *PIK3CA* and *PIK3R1* genes, coding for the p110α and p85α proteins, are prevalent in numerous tumor types as verified by deep sequencing analyses in the frame of projects such as TCGA [17–19]. Hotspots for PI3K gain-of-function mutations are found at the interface between the SH2 domains of p85 and the C2/helical structures in p110, thus leading to constitutive PI3K activation by releasing the inhibitory interactions between both subunits [13,40,42,43].

Here, we show that the key mechanistic events involved in pathologic PI3K hyperactivation, i.e. p85-driven plasma membrane recruitment and disruption of p85-imposed inhibition of p110, can be reproduced and traced in yeast. The key feature of reproducing in *S. cerevisiae* crucial aspects of PI3K regulation is that it provides an environment free of all the complex layers of regulation present in higher cells, but yet in an *in vivo* context. Thus, it permits studies of structure–function relationship properties inherent to the expressed proteins in the absence of additional inputs. However, limits to the use of the yeast system include the apparent lack of sensitivity to interaction with Ras, as well as its dependence on protein production and stabilization. When assaying catalytic p110 subunits in yeast in this and previous studies [20,21], we found that membrane targeting was essential for performance, by introduction of either an N-myristoylation or a C-terminal prenylation signal in the protein. The latter strategy was more effective, at least on p110α. We show here that a cluster of positively charged residues at the C2 domain (mutation C2/4KA) yields p110 inactive, but the effect of this multiple mutation can be by-passed by C-terminal prenylation. This confirms that the key function of the C2 domain is to facilitate contact with the negatively charged plasma membrane, as previously suggested [16,30]. However, prenylation of the regulatory subunit failed to activate the C2/4KA p110 mutant, indicating that these residues might also contribute to p85-dependent recruitment.

Among all class I PI3K catalytic isoforms individually tested, the p110α isoform appeared more active than the p110β isoform in terms of yeast growth inhibition, while p110γ and p110δ did not interfere with yeast growth when expressed alone. This could be related to a lower basal activity of the latter isoforms, as well as to a more limited ability to efficiently attach to membranes in the absence of the regulatory subunit, because when the oncogenic p85 truncation p65α–CAAX was co-expressed, all compatible class IA catalytic subunits (p110α, β, and δ) were equally and strongly activated.

By assaying p110 N-terminal truncations, we found that removal of the whole ABD domain does not eliminate intrinsic p110α and p110β PI3K activity, as long as the enzymes were artificially directed to the membrane. However, as expected, the ABD was necessary for p85-dependent activation, as p110α Δ1-107 did not respond to activation by p65α–CAAX. Nevertheless, these experiments are hampered by the difficulty in detecting by immunoblot the p110 N-terminal truncations, probably by compromised stabilization.

The artificial introduction of a C-terminal prenylation CAAX box in PI3K regulatory subunits mimicked in yeast its recruitment by tyrosine-phosphorylated RTKs in mammalian cells, causing an up-regulation of p110 activity by p65α–CAAX, or its down-regulation in the case of p85α–CAAX on membrane-driven Myr–p110α or p110β–CAAX. These results favor the notion that the regulatory interplay between p85 and p110 subunits can be achieved in yeast. Accordingly, mutations affecting the negatively charged p110α loop spanning residues 541–546, which interact with the positively charged p85 nSH2 domain [44], such as the clinically relevant E545K tested here, yielded Myr–p110α active even in the presence of p85α–CAAX. As reported by Miled et al. [36], the introduction of a K379E charge inversion mutant at the nSH2 domain in p85 reestablished the inhibitory interaction, thus suppressing gain-of-function of the p110α E545K mutant. The fact that this fine regulation can be reproduced in yeast illustrates that the heterologous model is instrumental, and that this particular regulatory event takes place in the absence of the multiple factors that regulate PI3K activity in higher cells.

Interesting mechanistic observations can also be inferred from the yeast model about performance of the most common p110α missense point mutation in clinical oncology, namely H1047R, mapping at the kinase domain [15,42]. First, the fact that it is the only p110 tested that is able to inhibit yeast growth by itself in the absence of membrane-targeting signals [21] favors the hypothesis that this mutation adopts a conformation that facilitates membrane recognition [45]. In this regard, the observation that the loss of positive charges at the C2 domain in this mutant suppresses the hypermorphic trait of the H1047R mutation in the combined p110α–C2/4KA–H1047R mutant reveals that its hyperactive conformation depends on a functional C2 domain. Furthermore, p110α H1047R activity in yeast was enhanced by both p65α–CAAX and p85α–CAAX, suggesting that it adopts a conformation that fails to respond to p85-imposed inhibitory interactions, but still responds to p85-driven membrane recruitment, in agreement with previous reports on the transformation activity of combined PI3K mutations in higher cells [46]. It has been shown that p-tyrosine peptides activate the p110α H1047R mutant, probably by disrupting p85-mediated inhibition and/or by increasing p110α lipid binding [45,47]. Although we cannot exclude the possibility that the yeast system is not sensitive to detect inhibition of p110α H1047R by p85α, our results suggest an essential contribution of membrane binding to the hyperactivity of p110α H1047R in cells, as previously suggested by structural analyses [44,48].

To validate the usefulness of our system on assessing the mechanism of putative pathogenic mutations in p85α, we chose a series of mutants described in the clinics. Among the mutations linked to cancer, two of them reproduced unequivocally the behavior pattern of p65, suggesting that they fully disrupted n/iSH2–helical/C2 inhibitory interactions. These were K379E, which introduce a negative charge at a key residue of the nSH2 domain [43,49], as discussed above and interestingly, N564D at iSH2. The latter point mutation, one of the most frequent *PIK3R1* found in tumors, is thought to disrupt the inhibitory interaction of the iSH2 domain in p85 with the C2 domain in p110 [12,30,45]. These results underscore the key involvement of the nSH2–helical and iSH2–C2 interactions in PI3K regulation. Interestingly, clinical mutations in nearby residues within the same hotspots, namely G376R (nSH2) and D560Y (iSH2) showed a different behavior: they maintained the ability to down-regulate membrane-targeted Myr–p110α, but they did activate soluble p110α. This might reflect a less potent gain-of-function activity than the previous class of mutations so that it can only be detected when p110 is not attached to the membrane by other means. In agreement with this view, G376R was less aggressive than K379E, and D560Y also seemed less potent as compared with N564D, regarding their ability to induce cell proliferation in higher cells, as tested by Sun et al. [43] and Jaiswal et al. [12] respectively. Other clinical mutations tested, ΔK459, predicted to disrupt the iSH2 α–helical fold [9,43] or R574T [12] did not behave differently than wild-type p85α in our system, which suggest that they may exert even a milder effect or, alternatively, that other cellular factors absent in yeast are involved in their oncogenic activity. This could be the case of the E666K mutation, mapping at the cSH2 domain, which is not presumably involved in p110 inhibition, as well as mutants in the SH3 (D13V) and GAP (N260S) domains of p85α that are assumed to contribute to pathology by mechanisms different than modulation of p110 activity. In line with this, germ line SHORT syndrome-related mutations in *PIK3R1*, which preferably target the cSH2 p85α domain, displayed in our yeast system an ability to modulate p110α indistinguishable from that of the wild-type p85α protein, even when large C-terminal truncations eliminating most of the cSH2 were assayed. This supports the idea that these mutations may cause pathology by different molecular mechanisms than those involved in cancer. The mutation R649W, the most common p85α variant associated with SHORT syndrome, causes impaired PI3K signaling, probably by defective association with tyrosine-phosphorylated protein effectors, such as IRS-1 [26,28,50]. Our results suggest that, unlike oncogenic gain-of-function mutants, p85α mutations associated with SHORT syndrome do not affect the inhibitory interaction between p85α regulatory and p110α catalytic subunits, but rather the interactions of the cSH2 p85α domain with other proteins, which are absent in our humanized yeast model. This provides a rationale for further scrutiny, both in our yeast model and in mammalian cells, which discriminates oncogenic and SHORT disease-associated mutations in *PIK3R1.*

## Materials and methods

### Strains, culture media, and growth conditions

All agar growth assays were performed with the *S. cerevisiae* YPH499 strain (*MATa ade2-101 trp1-63 leu2-1 ura3-52 his3-Δ200 lys2-801*). Growth conditions as well as serial dilution spot growth assays were described previously [20]. Halo growth assays were performed on the strain Y07202 (*MATa; his3Δ1; leu2Δ0; met15Δ0; ura3Δ0; YDR007w::kanMX4*) by using the 15e compound (PI3Kα inhibitor II Echelon®) as described previously [51].

### Plasmid construction and transformations

Transformation of *E. coli* and yeast and other basic molecular biology methods were carried out using standard methods. Plasmids pYES2 and pYES3/CT (Invitrogene), pYES2–GFP–Akt1, pYES2–GFP–Akt1 (K179M), pYES2–PTEN, pYES2–PTEN (C124S), YCpLG, YCpLG–p110α–CAAX, YCpLG–p110α (K802R)–CAAX, YCpLG–p110α, YCpLG–p110α (H1047R), YCpLG–p110α (E545K), YCpLG–p110β, and YCpLG–p110β–CAAX were already described [20,21]. YCpLG–Myr–p110α, YCpLG–MM–p110α, and YCpLG–FLAG–p110α were generated by PCR amplification of the first 1900 *PIK3CA* nucleotides from plasmids RCAS–Myr–p110α, RCAS–MM–p110α, and RCAS–FLAG–p110α [35] with primers containing *Bam*HI/*Xba*I sites at 5′ tails and then subcloned into plasmid YCpLG–p110α [21]. Plasmids YCpLG–p110α (C2/4KA)–CAAX (K410A/R412A/K413A/K416A), YCpLG–p110α (C2/4KA)–H1047R, YCpLG–p110α (C2/4KA), YCpLG–p110α (K227E)–CAAX, YCpLG–p110β (K230E)–CAAX, YCpLG–p110α (K227E)–H1047R, p110α (K227E), and YCpLG–Myr–p110α (E545K) were generated by introducing the corresponding mutations on the aforementioned plasmids as templates using site-directed mutagenesis with Turbo *Pfu*I DNA polymerase (Stratagene) and subsequent digestion with *Dpn*I according to the QuikChange^TM^ (Stratagene) recommendations.

Plasmids YCpLG–p110γ, YCpLG–p110γ–CAAX, YCpLG–p110δ, YCpLG–p110δ–CAAX, YCpLG–p110α(∆1-186), YCpLG–p110α(∆1-186)–CAAX, YCpLG–p110α(∆1-154)–CAAX, YCpLG–p110α(∆1-107), YCpLG–p110α(∆1-107)–CAAX, YCpLG–p110β(∆1-190), YCpLG–p110β(∆1-190-CAAX), YCpLG–p110β(∆1-117), and YCpLG–p110β(∆1-117–CAAX) (all with an N-terminal myc-tag) were constructed by subcloning into the *Xba*I/*Sal*I (p110γ, p110δ, and p110β truncation plasmids) or *Bam*HI/*Sal*I (p110α truncation plasmids) YCpLG linker sites the appropriate cDNAs, obtained from the corresponding pRK5 plasmids. The pRK5–Myc–p110 catalytic subunit and pRK5–Myc–p110 truncation plasmids will be described elsewhere, and were generated by cloning into pRK5 or pRK5–Myc the appropriate cDNAs, amplified by PCR using specific oligonucleotides and the corresponding plasmid templates: pCMVSPORT6 p110δ (mouse sequence) or pCMVSPORT6 p110γ (human sequence) (both from Mammalian Gene Collection, GE Dharmacon, Lafayette, CO, USA), or YCpLG–p110α or YCpLG–p110β (wt and -CAAX; containing an N-terminal myc-tag) [21]. Plasmids pYES3–p85α, pYES3–p85α–CAAX, pYES3–p65α, pYES3–p65α–CAAX, pYES3–p55α, pYES3–p50α, pYES3–p85β, pYES3–p65β, pYES3–p55γ, and pYES3–p101 (all with an N-terminal HA epitope) were constructed by subcloning into the *Eco*RI/*Xho*I pYES3 linker sites the appropriate cDNAs with *Eco*RI/*Sal*I flanking sites, obtained from the corresponding pRK5 plasmids. The pRK5–HA–PI3K-regulatory subunit plasmids will be described elsewhere, and were generated by cloning into pRK5 the appropriate cDNAs, amplified by PCR using specific oligonucleotides and the following plasmid templates: pCG–HA–p85α (mouse sequence; provided by A.C. Carrera, Centro Nacional de Biotecnología, Madrid, Spain), or pOTB7–p85β (human sequence), pCMVSPORT6–p55α (mouse sequence), pCMVSPORT6–p55γ (human sequence), and pCMVSPORT6-p101 (human sequence) (all from Mammalian Gene Collection, GE Dharmacon, Lafayette, CO, USA). Site-directed mutagenesis with the QuikChange^TM^ kit on pYES3–p85α–CAAX was performed in order to obtain the different p85α mutations. All the oligonucleotide sequences used in the present study are available upon request. When analyzing the sequences to verify the point mutations, we observed that the pCG–HA–p85α template used corresponded to the deposited sequence GenBank AAA39886.1, which bears a missense mutation as compared with the canonical NM_001077495.2 entry, involving a change of Glu^510^ to glycine (E510G). To verify that the presence of this amino acid change did not affect the validity of our results, we changed this residue back to glutamic acid by site-directed mutagenesis in our yeast expression vectors for HA–p65α and HA–p85α (both with and without the -CAAX signal) and tested them for their influence on yeast growth in co-expression with different p110 constructs used in the present study. The results obtained were totally unaffected by the E510G substitution in any case (results not shown).

### Immunoblots

Western blotting assays were carried out by standard techniques. Anti-G6PDH antibody (Sigma; 1:50000 dilution) was used as loading control. Monoclonal anti-HA antibodies (Clone 12CA5, Roche; 1:1000) were used as primary antibodies to detect the expression of the HA-tagged regulatory PI3K subunits.

## Abbreviations

ABD: adaptor-binding domain
cSH2: C-terminal SH2
GAP: GTPase-activating protein
IRS-1: insulin receptor substrate-1
iSH2: intermediate SH2
mTOR: mammalian target of rapamycin
nSH2: N-terminal SH2
PDK: phosphoinositide-dependent kinase
PI3K: phosphatidylinositol 3-kinase
PTEN: phosphatase and tensin homolog deleted on chromosome ten
RBD: Ras-binding domain
RTK: receptor tyrosine kinase
SHORT: short stature, hyperextensibility of joints and/or inguinal hernia, ocular depression, rieger anomaly, and teething delay
TCGA: The Cancer Genome Atlas
WT: wild type
